# What Are Patients Seeking When They Turn to the Internet? Qualitative Content Analysis of Questions Asked by Visitors to an Orthopaedics Web Site

**DOI:** 10.2196/jmir.5.4.e24

**Published:** 2003-10-10

**Authors:** Kristen S Shuyler, Kristin M Knight

**Affiliations:** ^1^University of WashingtonProgram for Educational Transformation Through TechnologySeattle WAUSA; ^2^University of WashingtonSchool of MedicineSeattle WAUSA

**Keywords:** Internet, information storage and retrieval, patients, orthopedics

## Abstract

**Background:**

More people than ever are turning to the Internet for health-related information, and recent studies indicate that the information patients find online directly affects the decisions they make about their health care. Little is known about the information needs or actual search behavior of people who use the Internet for health information.

**Objective:**

This study analyzes what people search for when they use a health-education Web site offering information about arthritis, orthopaedics, and sports-medicine topics. Additionally, it determines who is performing these searches: is it patients, friends or relatives of patients, or neither? Finally, it examines the similarities and differences among questions submitted by Web site visitors from different countries.

**Methods:**

Content analysis was performed on 793 free-text search queries submitted to a patient-education Web site owned and operated by the Department of Orthopaedics and Sports Medicine at the University of Washington Medical Center. The 793-query data set was coded into 3 schemes: (1) the purpose of the query, (2) the topic of the query, and (3) the relationship between the asker of the query and the patient. We determined the country from which each query was submitted by analyzing the Internet Protocol addresses associated with the queries.

**Results:**

The 5 most frequent reasons visitors searched the Web site were to seek: (1) information about a condition, (2) information about treatment, (3) information about symptoms, (4) advice about symptoms, and (5) advice about treatment. We were able to determine the relationship between the person submitting the query and the patient in question for 178 queries. Of these, the asker was the patient in 140 cases, and the asker was a friend or relative of the patient in 38 cases. The queries were submitted from 34 nations, with most coming from the United States, Australia, the United Kingdom, and Canada. When comparing questions submitted from the United States versus those from all other countries, the 3 most frequent types of questions were the same for both groups (and were the top 3 question types listed above).

**Conclusions:**

These results provide the University of Washington Department of Orthopaedics and Sports Medicine, as well as other organizations that provide health-information Web sites, with data about what people around the world are seeking when they turn to the Internet for health information. If Web site managers can adapt their health-information Web sites in response to these findings, patients may be able to find and use Internet-based health information more successfully, enabling them to participate more actively in their health care.

## Introduction

Millions of people are turning to the Internet for health-related information, and the information they find often directly affects the decisions they make about their health care. A recent study estimated that 73 million Americans, or 62% of Americans with access to the Internet at the time, have used the Internet to search for medical information or other information relevant to their health care [1]. Another study estimated that 12.34 million health-related Web searches are conducted worldwide every day [2]. People often turn to the Internet for health and medical information because of its convenience and for the opportunities it provides for communication [3]. Many people use the Internet to supplement the information given to them by their physician [1,4], to become more involved in their health care decisions [5], or to become more educated about their condition or the condition of a friend or relative.

The medical information that people find on the Internet may affect their health care decisions, health status, and mental status. Two recent studies reported on Americans who have searched the Internet for medical information, termed "health seekers." Ninety-two percent of health seekers reported that the health information they obtained during their last Internet search was useful and relevant [4], and 68% reported that the medical information they found on the Internet played an influential role in the health care decisions they made for themselves or their loved ones [1]. In 2002, 62% of health seekers said the information they found online improved how they cared for themselves, up from 48% in 2000 [1]. People may be able to meet some of their psychological needs related to their health care by using Internet-based tools to answer their questions [3]. Interacting with others online may also lead to improved health status and decreased health care utilization, according to a recent study that examined e-mail discussion groups [5].

Research suggests that the Internet has revolutionized the way patients access health care information, learn more about their conditions, and make health care decisions. Little data exists, however, about the ways that patients actually use the Web [6,7], or about patients' information needs when they use the Internet, such as the "reasons behind online information-seeking" and the "behavior of health users" on the Internet [6]. To explore patients' information needs when they consult the Internet, this study analyzes what people search for when they use a patient-education Web site operated by the Department of Orthopaedics and Sports Medicine at the University of Washington Medical Center. Additionally, it determines who is performing these searches, and it compares questions submitted by Web site visitors from around the world.

Recent studies asking similar questions have analyzed the topics of e-mails sent to doctors [8,9] and the data from surveys asking about patients' Web searches for medical information [10]. Our study builds on this previous work by investigating the actual searches performed by one specific set of Internet users—those who visit the University of Washington Department of Orthopaedics and Sports Medicine Web site. The University of Washington Department of Orthopaedics and Sports Medicine, as well as other organizations providing health information Web sites, can use these data about these users to develop their understandings of the information needs of patients in general, and to improve their Web sites to provide effective educational materials for their users. In turn, patients who are knowledgeable about their health can participate more actively with their physicians in determining a health care plan that is best suited for them.

## Methods

**Figure 1:**
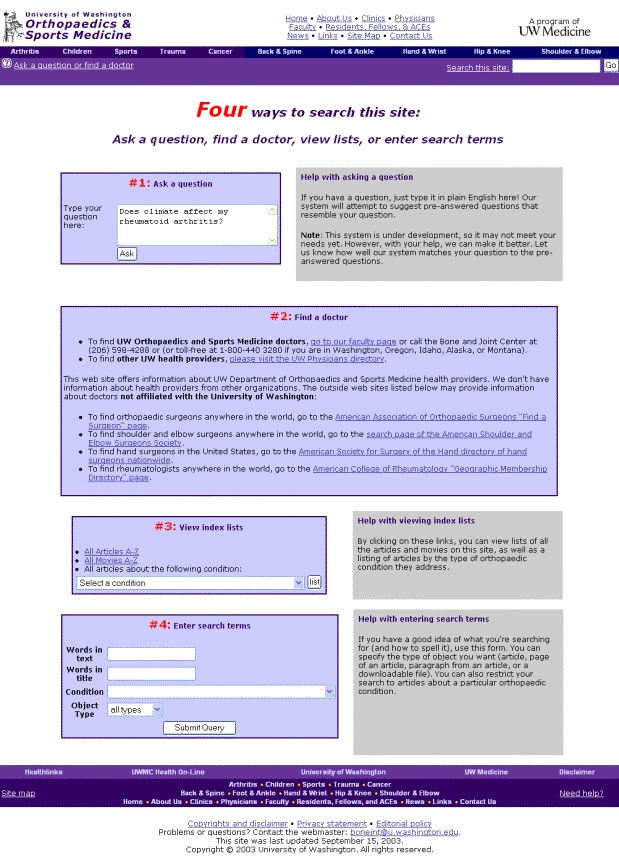
Web site search-box page

**Figure 2:**
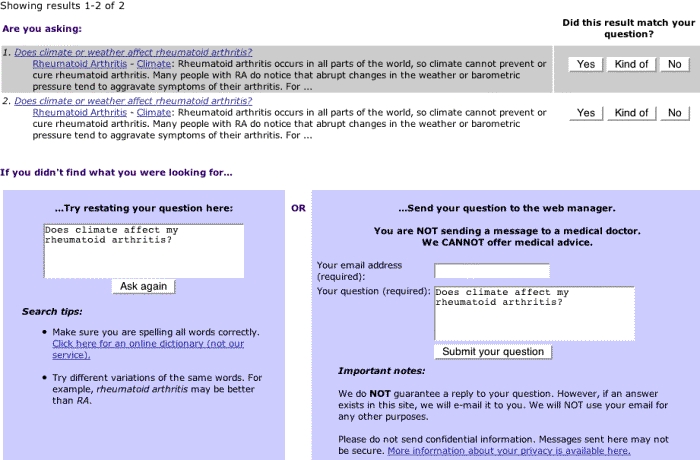
Web site search-results page

Content analysis was performed on free-text queries submitted to the Web site of the University of Washington Department of Orthopaedics and Sports Medicine [11], which features multimedia information on arthritis, orthopaedics, and sports-medicine topics. The Web site, which has been operating since 1995, provides articles and videos on over 200 topics to an average of 4000 visitors a day worldwide. The goal of the Web site is "to offer patient-education materials that support users' self-directed learning and help them answer their questions" [12]. In addition to browsing the Web site, visitors can query it in 3 ways: (1) by conducting a simple keyword search, (2) by e-mailing the Web site manager, or (3) by searching the Web site with the *Ask a Question* function (see Figure 1 and Figure 2 for screenshots of the search box page and the search results page). This search function was built specifically for this Web site, after informal analysis of searches performed in the keyword box revealed a high number of long queries resembling questions. This study analyzes the queries users submitted to the Ask a Question function, because that function encourages the users to describe what they are looking for in more detail than the small keyword search box does.

When using the Ask a Question function, users type free-text questions into a search box that is large compared to typical keyword search boxes. A simple computer program built for the Ask a Question function parses these free-text questions to make them compatible with the program's search algorithm. The users receive search results consisting of similar questions to which the Web site already offers answers. They can then choose which question/answer is the most relevant or similar to their information need. Implemented in November 2001, the Ask a Question system handles more than 1000 questions each month.

### Sample

The questions examined in this study were obtained by a systematic sample of all the free-text queries submitted to the Web site's Ask a Question function during March and June of 2002.

March and June were selected so the data set would consist of recent queries. May was omitted because the queries from that month were used to develop the coding scheme. April was omitted because it had an unusually-high number of submissions due to a campus event related to the Web site.

All search queries submitted during March and June were retrieved from the Web site's log files. We also obtained the date and time each query was submitted and the Internet Protocol (IP) address (the unique numeric address of the computer from which each query was submitted [13]).From the IP address, we were able to determine the country of origin of each submission.

In March and June, respectively, 2290 and 2208 queries were submitted to the site. These queries were imported into Excel, sorted by IP address and time, and examined. Identical questions submitted from the same IP address were deleted, as were extremely-similar (but not identical) questions submitted from the same IP address within a period of 24 hours. Queries submitted with no text and those submitted from askers who were not patients nor were asking for patients were deleted. Examples of queries from people who were not patients nor asking for patients include queries from students, queries of a derogatory or crude nature, or queries that did not pertain to the Web site or its contents in any way. Queries submitted by staff members when testing the site were deleted. After these deletions were made, the resulting data set consisted of 885 and 702 queries for March and June, respectively, for a total of 1587 queries.

To obtain the sample on which our analyses were conducted, the queries were sorted by the date and time of submission (oldest to most recent) and every other query was selected (in March we started with the first query and in June we started with the second). This systematic sampling method was used so that queries would be selected from the full range of the month, from start to finish, to account for any possible changes in visitors' behavior over this period (eg, if people respond to the beginning of a month with a renewed interest in their health and therefore ask different types of questions). The resulting sample consisted of 793 queries.

### Content Analysis and Coding System

Because the underlying meanings of the questions submitted to the Web site were of interest (what users actually wanted to find, as opposed to the use of certain words or the number of words per query), a qualitative, descriptive approach was selected. We analyzed the queries by reading them and looking for themes and patterns, then developing a coding system to describe the data. These procedures are typical steps in content analysis. We decided content analysis was useful for this project because it is an unobtrusive analysis method, it can handle material that is not structured (and thus can tolerate free-text queries), and it can easily accommodate large amounts of data [14]. Content analysis has been utilized to investigate patients' needs and concerns in medical studies [15,16,17], but we have not found its use in studies of online health-information needs.

#### Development of Coding System

To develop the coding system, a test set of queries submitted to the Ask a Question function during the month of May 2002 was retrieved from the Web site's log files and imported into Excel. After conducting the same process of deletions as described above, the resulting 951 queries were used to develop the coding scheme. The submissions were examined and a preliminary code was developed to reflect the overall trends observed in the data. As additional questions were examined and applied to the code, the coding system was altered and refined. During this process, the code was shared with a physician and a registered nurse, both of whom had extensive research experience, to obtain their feedback. The result was a coding system that was clear, concise, and descriptive of all the available data in the test set.

The coding system we developed consisted of 3 measures: (1) the asker's purpose in submitting the query, (2) the main topic addressed in the query, and (3) the relationship between the asker and patient. For the first measure, the asker's purpose in submitting the query, the 5 coding options included: (1) the asker was seeking advice or an opinion, (2) the asker was seeking an interpretation of information they already possessed, (3) the asker was seeking information (includes keyword searches performed using the Ask a Question function), (4) the asker was solely sharing information, and (5) the asker's purpose in submitting the question was unclear, or it didn't fit into any of the other categories. See Table 1 for examples of the 5 coding options.

The second measure, the main topic addressed in the question, consisted of 5 coding options: (1) questions with a main topic pertaining to physicians, support groups, or other resources, (2) questions asking about conditions (including syndromes), symptoms, and/or injuries, (3) questions that addressed treatments (including procedures), tests/labs, the process of diagnosis, prognosis, prevention, diet, exercise, medication, and/or lifestyle, (4) questions regarding anatomical structures, and (5) questions in which the main topic was unclear or did not fit into any of the above categories (see Table 1 for examples of the 5 coding options).

The third measure addressed the relationship between the asker and the patient in the question. The 3 codes were: (1) the asker self-identified as the patient, (2) the asker self-identified as a relative or friend of the patient, and (3) the relationship between the asker and patient could not be determined with the information provided (see Table 1 for examples of the 3 coding options).

**Table 1:** Coding options for 3 content analysis measures

**Coding Options for Purpose of Query**	**Example From Data Set**
(1) Asker seeking advice or opinion	What should I eat if I have back pain?
(2) Asker seeking an interpretation	What does it mean when a neurosurgeon says 'You have a broken back'?
(3) Asker seeking information	How do you get arthritis?
(4) Asker is sharing information	I have rheumatoid arthritis.
(5) Purpose is unclear	Human growth hormone volunteer.
**Coding Options for Main Topic of Query**	**Example From Data Set**
(1) Physicians, support groups, and/or resources	Who is a good surgeon in Memphis for rotator cuff surgery?
(2) Conditions, symptoms, and/or injuries	What is compartmental syndrome?
(3) Treatments/procedures, tests/labs, process of diagnosis, prognosis, prevention, diet, exercise, medication, and/or lifestyle	How is arthritis diagnosed?
(4) Anatomical structures	What is a rotator cuff?
(5) Unclear or does not belong in 1-4	Why are shorter people quicker than taller people besides artheritis [*sic*]?
**Coding Options for Relationship of Asker to Patient in Question**	**Example From Data Set**
(1) Asker is patient	What is my life expectancy with RA?
(2) Asker is a relative/friend of patient	How does my daughter avoid multiple shoulder dislocations?
(3) Relationship unknown	How does one know if the rotator cuff is injured or torn?

#### Inter-rater Reliability of Coding System

The reliability of this coding system was determined before it was applied to the actual 793-query data set. A sample of the actual data set (queries submitted during the months of March and June) was created using a systematic sampling technique. With the data sorted by date and time (oldest to most recent), every twentieth query was selected, starting with the second, creating a 40-query subset of the data set. Four coders applied the coding system to the 40-query sample. Besides the 2 authors, the coders included 2 individuals who were familiar with the Web site but not involved with this project. We introduced both people to the coding scheme and gave them the same instructions. Each coder then coded the pilot sample on the same day in a similar setting. The results were collected, entered into an Excel spreadsheet, and imported into SPSS (Statistical Package for Social Sciences). Fleiss' kappa statistical method was used to determine the inter-rater agreement for each of the 3 measures described above. Fleiss' kappa statistic is a measurement of inter-rater agreement among more than 2 raters when the coding is on a categorical scale [15]. For the first 2 measures (purpose and main topic of query), moderate agreement was achieved (κ = 0.679 and κ = 0.697, respectively) [18]. For the third measure (relationship between asker and patient), strong agreement was achieved (κ = 0.763) [18].

#### Application of Coding System

Once the inter-rater reliability of the coding system was determined, the second author applied it to the 793-query data set, one measure at a time. Because the first measure, purpose of the query, was of the most interest to us, the same author coded it twice to determine intra-rater reliability, using Cohen's kappa statistic. Of the 793 entries coded twice by the same author, 29 discrepancies were found, resulting in a high level of agreement (κ = 0.915). The discrepancies were most often between the purposes "seeking advice" and "seeking information." The authors discussed the discrepancies and came to a unanimous decision on each one; in the process, the codes "seeking advice" and "seeking information" were further clarified. An inquiry was deemed to have the purpose of seeking information when the response elicited would not be need to be based on individual circumstances (ie, the response would be the same for multiple askers), whereas an inquiry that required a personalized response based on a patient's unique situation was categorized as asking advice.

After the "purpose" code had been applied to the data, the second author coded the data for the second measure, the main topic addressed in the query. All of the questions were then grouped by the code they received, examined in detail, and further categorized. For instance, all of the submissions that received a code of "1" pertained to physicians, support groups, and/or resources. After receiving the code of "1," they were further categorized by the specific topic they addressed (eg, physicians *or* support groups *or* resources).

Next, the data were coded for the third measure, the relationship between the asker and the patient in the question. The first code was assigned only when the asker explicitly identified himself/herself as the patient (eg, How should I treat my arthritis?). The second code was assigned only when the asker made it clear that they were asking for someone else (eg, My daughter was diagnosed with arthritis. How can I learn more about it?). The remaining queries were assigned the third code.

## Results

### Purpose of Queries

The most frequent purpose of the 793 queries was to seek information (73%). This was followed by seeking advice (23%), sharing information (3%), and seeking an interpretation (1%). For 5 of the questions (1%), the purpose was unclear (see Table 2).

**Table 2:** Purposes of queries

**Purpose**	Frequency (%)*†
Seeking information	576 (73%)
Seeking advice	182 (23%)
Sharing information	22 (3%)
Seeking an interpretation	11 (1%)
Unclear	5 (1%)
Total	793

^*^ The total values exceed 100% as 3 queries had 2 or more purposes.

^†^ Percentages do not add up correctly due to rounding.

### Topic of Queries

Topics were determined for those queries with the 3 most frequent purposes (seeking information, seeking advice, or sharing information; a total of 780 queries). See Table 3 for the topics about which visitors to the site inquired and their frequencies.

**Table 3:** Topics of queries

**Topic**	Frequency (%)*†
Condition	247 (32%)
Treatment	164 (21%)
Symptoms	131 (17%)
Medication	42 (5%)
Injury	40 (5%)
Physician	33 (4%)
Anatomy	24 (3%)
Exercise	22 (3%)
Tests/Labs	20 (3%)
Diet	10 (1%)
Prognosis	10 (1%)
Diagnosis	6 (1%)
Lifestyle	5 (1%)
Resources	5 (1%)
Prevention	4 (1%)
Support groups	1 (0%)
Unclear	40 (5%)
Total	780

^*^ The total values exceed 100% as 20 queries had 2 or more topics.

^†^ Percentages do not add up correctly due to rounding.

Of the 576 submissions with the purpose of seeking information, the most frequent topic was a condition (39%). Treatment was also a frequent topic (20%), followed by symptoms (11%) (see Table 4).

Of the 793-query dataset, 182 were seeking advice. The most frequent topic about which users sought advice was symptoms (28%). Other frequent topics for which advice was sought were treatment (26%), medication (12%), a condition (10%), and injury (6%) (see Table 4).

**Table 4:** Specific topics: seeking information, seeking advice, sharing information

**Topic**	**Frequency (%)** of Queries Seeking Information*†	**Frequency (%)** **of Queries Seeking Advice**†‡	**Frequency (%)** **of Queries Sharing Information**†
Condition	227 (39%)	18 (10%)	2 (9%)
Treatment	114 (20%)	48 (26%)	3 (14%)
Symptoms	64 (11%)	51 (28%)	16 (73%)
Injury	30 (5%)	10 (6%)	1 (5%)
Physician	25 (4%)	9 (5%)	0
Anatomy	24 (4%)	1 (1%)	0
Medication	22 (4%)	21 (12%)	0
Tests/Labs	14 (2%)	6 (3%)	0
Exercise	13 (2%)	9 (5%)	0
Diagnosis	6 (1%)	0	0
Diet	6 (1%)	4 (2%)	0
Resources	5 (1%)	0	0
Prognosis	4 (1%)	6 (3%)	0
Prevention	3 (1%)	1 (1%)	0
Lifestyle	1 (0%)	4 (2%)	0
Support groups	1 (0%)	0	0
Unclear	33 (6%)	3 (2%)	0
Total	576	182	22

^*^ The total values exceed 100% as 14 queries had 2 or more topics.

^†^ Percentages do not add up correctly due to rounding.

^‡^ The total values exceed 100% as 8 queries had 2 or more topics.

Twenty-two of the 793 questions had a sole purpose of sharing information. Of these, 16 (73%) were sharing about symptoms and 3 (14%) were sharing about treatment (see Table 4).

**Table 5:** Most frequent topics by most frequent purposes

**Seeking Information**	**Seeking Advice**	**Sharing Information**
(1) Condition	(1) Symptoms	(1) Symptoms
(2) Treatment	(2) Treatment	(2) Treatment
(3) Symptoms	(3) Medication	(3) Condition
(4) Injury	(4) Condition	(4) Injury
(5) Physician	(5) Injury	

**Table 6:** Five most frequent query types

**Inquiry**	**% (n=793)**
(1) Seeking information about a condition	29%
(2) Seeking information about treatment	14%
(3) Seeking information about symptoms	8%
(4) Seeking advice about symptoms	6%
(5) Seeking advice about treatment	6%

#### Summary of Topics

By examining the topics of the questions with the purposes of seeking information, seeking advice, or sharing information, we determined the most frequent types of queries submitted to the Web site (see Table 5 and Table 6). They were:

- Seeking information about a condition
- Seeking information about treatment
- Seeking information about symptoms
- Seeking advice about symptoms
- Seeking advice about treatment

### Relationship Between Asker and Patient

Of the 793 queries in the dataset, only 178 (22%) provided enough information for the researchers to determine the relationship between the asker and the patient. Of the 178 entries for which the relationship could be deduced, there were 140 entries (79%) in which the individual posing the question was the patient (ie, the askers were asking for themselves) and 38 entries (21%) in which the asker was clearly asking for someone else (eg, a mother, daughter, or friend).

**Table 7:** Purposes of queries by relationship with patient

**Purpose**	**Frequency (%)** of Queries When Asker Was the Patient*†	**Frequency (%)** of Queries When Asker Was Friend/Relative of the Patient*†	**Frequency (%)** **of Queries When Relationship of Asker and Patient Could Not Be Determined**
Seeking advice	96 (69%)	20 (53%)	77 (13%)
Seeking information	25 (18%)	17 (45%)	516 (85%)
Sharing information	16 (11%)	1 (3%)	5 (1%)
Seeking an interpretation	4 (3%)	1 (3%)	7 (1%)
Total	140	38	605

^*^ The total values exceed 100% as 1 query had 2 or more purposes.

^†^ Percentages do not add up correctly due to rounding.

**Table 8:** Topics about which advice was sought

**Topic**	**Frequency (%)** of Queries Asking Advice About Specific Topics When Asker Was the Patient in Question*†	**Frequency (%)** **of Queries Asking Advice About Specific Topics When Asker Was Relative/Friend of the Patient in Question**†‡
Symptoms	33 (34%)	3 (15%)
Treatment	22 (23%)	8 (40%)
Medication	10 (10%)	3 (15%)
Condition	9 (9%)	2 (10%)
Injury	6 (6%)	0
Physician	5 (5%)	2 (10%)
Lifestyle	4 (4%)	0
Exercise	3 (3%)	1 (5%)
Prognosis	3 (3%)	1 (5%)
Tests/Labs	3 (3%)	1 (5%)
Diet	2 (2%)	0
Anatomy	1 (1%)	0
Prevention	0	1 (5%)
Unclear	1 (1%)	1 (5%)
Total	96	20

^*^ The total values exceed 100% as 6 queries had 2 or more topics.

^†^ † Percentages do not add up correctly due to rounding.

^‡^ ‡ The total values exceed 100% as two queries had 2 or more topics.

Of the 140 entries in which the asker was the patient, 69% of the time the asker was seeking advice. Other purposes included seeking information (18%), sharing information (11%), and seeking an interpretation (3%) (see Table 7). Of the 96 cases in which the askers were seeking advice, the most frequent topic was symptoms (34%). Other topics included treatment (23%), medication (10%), a condition (9%), and injury (6%) (see Table 8). Of the 25 cases in which the askers were seeking information, they were most frequently inquiring about treatment (44%), symptoms (16%), a condition (12%), and exercise (8%) (see Table 9).

**Table 9:** Topics about which information was sought

**Topic**	**Frequency (%)** of Queries Asking for Information About Specific Topics When Asker Was the Patient in Question*†	**Frequency (%)** **of Queries Asking for Information About Specific Topics When Asker Was Relative/Friend of the Patient in Question**†‡
Treatment	11 (44%)	3 (18%)
Symptoms	4 (16%)	1 (6%)
Condition	3 (12%)	9 (53%)
Exercise	2 (8%)	0
Anatomy	1 (4%)	2 (12%)
Diagnosis	1 (4%)	0
Diet	1 (4%)	0
Injury	1 (4%)	0
Lifestyle	1 (4%)	0
Medication	1 (4%)	1 (6%)
Physician	1 (4%)	3 (18%)
Prevention	1 (4%)	0
Support groups	1 (4%)	0
Resources	0	1 (6%)
Unclear	1 (4%)	0
Total	25	17

^*^ The total values exceed 100% as 4 queries had 2 or more topics.

^†^ Percentages do not add up correctly due to rounding.

^‡^ The total values exceed 100% as two queries had 2 or more topics.

Of the 38 entries in which the asker was a relative or friend of the patient, the 2 main purposes were seeking advice (53%) and seeking information (45%) (see Table 7). Of the 20 cases in which advice was sought, 40% were seeking advice about treatment, 15% about medication, and 15% about symptoms (see Table 8). In 17 cases, askers who were inquiring for a relative or friend were seeking information. Most frequently, they were seeking information about a condition (53%). Other frequent topics were physicians (18%), treatment (18%), and anatomy (12%) (see Table 9).

#### Summary of Relationship Between Asker and Patient

In the cases in which the askers were submitting questions relevant to the health of their friends, their relatives, or themselves, advice was the most frequent type of query. When the relationship between the asker and the patient could not be deduced, information was the most frequent type of query. Table 10 compares the 4 most frequent types of inquiries posed by (1) askers who were the patient or relatives/friends of the patient in the question and (2) askers whose relationship to the patient could not be determined.

**Table 10:** Four most frequent types of queries by relationship

**Asker was Patient or Relative/Friend of Patient in Question**	**Relationship Between Asker and Patient Could Not Be Determined**
(1) Seeking advice about symptoms	(1) Seeking information about a condition
(2) Seeking advice about treatment	(2) Seeking information about treatment
(3) Seeking information about treatment	(3) Seeking information about symptoms
(4) Seeking advice about medication	(4) Seeking information about an injury

### Country of Origin

The queries considered in this study originated from 34 nations. Most were from the United States (647 queries, or 82%). Other countries originating a large number of queries included Australia (38 queries), the United Kingdom (34 queries), and Canada (22 queries). India and New Zealand were the source of 6 questions each, and 3 were from South Africa. Two queries originated from each of the following: Argentina, France, Germany, Ireland, Macedonia, Malaysia, Netherlands, Pakistan, Singapore, and Turkey. One query was submitted from each of the following: Belgium, Brazil, Colombia, Egypt, Hong Kong, Israel, Jamaica, Mexico, Papua New Guinea, Peru, Saudi Arabia, Spain, Sweden, Switzerland, Syria, Trinidad and Tobago, and Venezuela. Because of the relatively small number of queries from countries other than the United States, we divided the data into 2 sets: questions sent from the United States, and questions sent from all other nations.

Of the 647 queries from the United States, most were submitted for the purpose of seeking information (71%) or advice (24%) (see Table 11). Of the 458 cases in which the purpose was seeking information, 39% of the time information was sought about a condition. Other frequent topics were treatment (20%) and symptoms (11%) (see Table 12). Of the 155 cases in which the purpose was seeking advice, 30% of the questions sought advice about symptoms. Other frequent topics were treatment (23%), medication (11%), and a condition (9%) (see Table 13).

**Table 11:** Purposes of queries submitted by location of query submission

**Purpose**	**Frequency (%)** of Queries Submitted From the United States*†	**Frequency (%)** **of Queries Submitted From Countries Other Than the United States**†‡
Seeking information	458 (71%)	118 (81%)
Seeking advice	155 (24%)	27 (19%)
Sharing information	21 (3%)	1 (1%)
Seeking an interpretation	11 (2%)	0
Unclear	4 (1%)	1 (1%)
Total	647	146

^*^ The total values exceed 100% as two queries had 2 or more purposes.

^†^ Percentages do not add up correctly due to rounding.

^‡^ The total values exceed 100% as 1 query had 2 or more purposes.

**Table 12:** Topics about which information was sought by location of query submission

**Topic**	**Frequency (%)** of Queries From the United States*†	**Frequency (%)** **of Queries From Outside the United States**†‡
Condition	179 (39%)	48 (41%)
Treatment	92 (20%)	22 (19%)
Symptoms	51 (11%)	13 (11%)
Physician	25 (6%)	0
Injury	24 (5%)	6 (5%)
Anatomy	18 (4%)	6 (5%)
Medication	17 (4%)	5 (4%)
Tests/Labs	12 (3%)	2 (2%)
Exercise	7 (2%)	6 (5%)
Diagnosis	5 (1%)	1 (1%)
Diet	5 (1%)	1 (1%)
Resources	4 (1%)	1 (1%)
Prevention	3 (1%)	0
Prognosis	3 (1%)	1 (1%)
Support groups	1 (0%)	0
Lifestyle	0	1 (1%)
Resources	0	1 (1%)
Unclear	25 (6%)	8 (7%)
Total	458	118

^*^ The total values exceed 100% as 11 queries had 2 or more topics.

^†^ Percentages do not add up correctly due to rounding.

^‡^ The total values exceed 100% as 3 queries had 2 or more topics.

**Table 13:** Topics about which advice was sought by location of query submission

**Topic**	Frequency (%) of Queries From the United States*	**Frequency (%) of Queries from Outside the United States**†
Symptoms	47 (30%)	4 (15%)
Treatment	36 (23%)	12 (44%)
Medication	17 (11%)	4 (15%)
Condition	14 (9%)	4 (15%)
Injury	9 (6%)	1 (4%)
Physician	9 (6%)	0
Exercise	8 (5%)	1 (4%)
Tests/Labs	6 (4%)	0
Prognosis	5 (3%)	1 (4%)
Lifestyle	4 (3%)	0
Diet	3 (2%)	1 (4%)
Prevention	1 (1%)	0
Unclear	3 (2%)	0
Total	155	27

^*^ The total values exceed 100% as 6 queries had 2 or more topics.

^†^ The total values exceed 100% as two queries had 2 or more topics.

Of the 146 submissions from all countries other than the United States, the most frequent purposes were seeking information (81%) and seeking advice (19%) (see Table 11). Of the 118 cases in which the askers were seeking information, they were most frequently asking about a condition (41%). Other frequent topics included treatment (19%) and symptoms (11%) (see Table 12). Of the 27 cases in which the askers were seeking advice, they were most often seeking advice about a treatment (44%). Other frequent topics included condition (15%), medication (15%), and symptoms (15%) (see Table 13).

#### Summary of Country of Origin

The 3 most frequent types of inquiries submitted from the United States and from all other countries were the same (see Table 14). They were:

- Seeking information about a condition
- Seeking information about treatment
- Seeking information about symptoms

The chi-square test was used to evaluate these variables, and there were no statistically significant differences in types of questions asked based on location (Ï ^2^_2_= 0.16, *P*= .92).

## Discussion

These results indicate that when people turn to the University of Washington Orthopaedics and Sports Medicine Web site with health questions, their most frequent motives are to find information about a condition, treatment, or symptoms. In our data set, conditions, treatments, and symptoms were the most frequent topics whether the purpose of the query was to seek information, seek advice, or share information. These results are similar to those of other studies suggesting that that the most frequent reasons people use the Internet for medical information are to seek information and to seek advice about topics such as conditions, treatments, and symptoms [8,10].

One similar study, conducted by Eysenbach et al, analyzed 209 unsolicited e-mails sent to physicians through a university hospital Web site, and found that 34% of the e-mails contained requests for general information about a condition or disease, while 75% of the e-mails contained specific questions, most frequently pertaining to treatment options, specialist referrals, alternative treatments, and whether a condition was curable [8]. The main topics asked by Web site users in our study were similar to those asked in Eysenbach's study. Conditions and treatments were the 2 most frequent topics in our study, while physicians and prognosis were present though less frequent (see Table 3). However, our findings differed from Eysenbach's with respect to the "purpose" of the requests: of the 793 search queries analyzed in our study, 73% were asking for information, and 23% were seeking advice (see Table 2). This variation is likely due to the different types of questions analyzed in the 2 studies. In Eysenbach's study, the objects of analysis were e-mails that patients sent to the physicians responsible for the Web site. Our study, on the other hand, analyzes questions that were submitted as a search method on a Web site, not questions that were sent in e-mail messages. Thus, we are analyzing what patients seek when they are anonymously searching a health information Web site, rather than what they seek when they make the effort to formulate a question and e-mail it to the Web site owners.

Another study with similar results as ours (but different methods) was conducted by O'Connor et al [10]. O'Connor's study investigated the information that patients of a gastroenterology clinic sought when using the Internet. By analyzing questionnaires that patients filled out when they visited the clinic, the researchers found that of the 462 patients who reported having access to the Internet, 51% had used the Internet within the last year to search for medical information. Of the patients who had utilized the Internet for medical information, 31% sought general disease information, 23% sought information about treatment options, 18% inquired about medications, 14% sought information about diet and nutrition, and 10% inquired about alternative medicine. Our results show similar trends: 32% of the queries in our study asked about a condition or disease, 21% asked about treatment, 5% asked about medications, and 1% asked about diet (see Table 3). However, because the data in O'Connor's study were derived from responses to questionnaires, their results describe what patients perceive and report about their Internet use, which may differ from their actual behavior [2,7,19]. In contrast to the study by O'Connor et al, our study examines the log files of a health information Web site to analyze the *actual* questions that Web site visitors ask, rather than relying on patients to report their behavior while at the doctor's office.

The results of our study suggest that people searching the Internet for medical information that pertains to their health (or the health of a friend or relative) may have different information needs than users searching for medical information that does not apply to their health (or the health of a friend or relative). The inquiries submitted by askers who identified themselves as the patient in question (or a friend or relative of the patient in question) did not follow the overall trend of seeking information. As shown in Table 10, when the relationship between the asker and patient could be deduced, the most frequent purpose of the inquiry was to seek *advice*, whereas when the relationship between asker and patient could not be determined, the most frequent purpose was to seek *information*.

These results also suggest that Web site visitors from around the world have similar goals when turning to the Internet for health-related information. The 3 most frequent types of inquiries submitted from the United States were not statistically different from those submitted from all other countries combined ( *P*= .92) (see Table 14). Thus, among *all* the Web site visitors from around the world, the most frequent searches performed were to seek information about a condition, treatment, or symptoms. These results indicate that the global audience of the University of Washington Orthopaedics and Sports Medicine Web site has similar information needs, regardless of geography.

**Table 14:** Three most frequent types of queries by location of submission (frequency of query)

**Query type**	**United States** **(n=647)**	**Countries Other Than the United States** **(n=146)**
Seeking information about a condition	179	48
Seeking information about treatment	92	22
Seeking information about symptoms	51	13

The results of this study will inform the future development, design, and content of the University of Washington Orthopaedics and Sports Medicine Web site. The Web site managers aim to improve the site's usefulness, and this data about the users' information behavior and needs will help the managers improve it specifically for the Web site's audience. It appears that the focus of the Web site is already in line with the main needs of its users, but this study has identified several areas in which user interest outstrips the Web site's coverage. First, although there are many articles about various conditions, the Web site can be improved in the areas of treatment and symptom information for orthopaedic conditions, which were the second and third most frequent topics for all types of questions. Second, "treatment" in general was the second most frequent topic asked about by users, but the Web site focuses on surgical interventions and offers few articles on nonsurgical treatments. Thirdly, the Web site does not yet provide any articles that help patients learn more about specific symptoms. Of the queries that were seeking advice, symptoms were the most frequent topic. This suggests that the articles focusing on symptoms might need to be written differently than articles about conditions or treatments (which were the most frequent topics when users sought information). Another way to respond to the findings of this study might be to redesign the Web site's user interface to reflect the topics for which the users search most often. The current design features 10 main labels: 5 body parts (eg, "shoulder & elbow") and 5 orthopaedic specialties (eg, "rheumatology"). Based on this study's findings, the Web site designers are considering changing the user interface to feature labels that are more in line with the topics for which its users search, such as "conditions," "treatments," and "symptoms." These are just a few of the ways that the Web site can be adapted to better meet the needs of its users, based on this data about what patients are really interested in learning.

This study has several possible limitations. First, the development of the coding system and the coding of the queries were performed by the same person. Krippendorf [14] suggests that it is less than ideal for the developer of a code to also be the administrator; however, he concedes that it is acceptable when resources are limited. We determined the inter-rater and intra-rater reliability measures to diminish and be aware of the possible effects of this limitation.

A second limitation is that the inter-rater reliability was determined by coding queries according to their broad topic category (refer to Table 1) rather than by specific category. Once moderate agreement was achieved with respect to the broad topic categories (eg, "condition, symptoms, and/or an injury"), the lead author went a step further to determine each query's *specific* topic (eg, "injury"). However, no inter-rater or intra-rater reliability measures were determined for this step.

A third limitation of this study pertains to the specific set of Internet users considered. Because our data consisted of questions submitted to a health education Web site focused on arthritis, orthopaedics, and sports-medicine information, most individuals submitting the questions were probably interested in information relating to these topics. Therefore, it may be difficult to use these results to make claims about how people in general use the Internet to search for any type of medical information. Moreover, this study focused on user information needs based on search queries, which excludes people who browse the Web site instead of searching it.

A fourth limitation is a common drawback of log-file analysis. When examining the IP addresses of the computers used to submit questions, we cannot know if the people who submitted the questions were actually from the countries in which their computers were operating. For example, questions from computers not based in the United States could have been American travelers or Americans who choose to live abroad, just as questions from United States-based computers could be from exchange students, travelers, or other people not from the United States. Thus, the distinction made between questions from United States-based askers and those from other countries may not have reflected the actual identity of the askers.

### Conclusions

The results of this study are important to the improvement of the University of Washington Orthopaedics and Sports Medicine Web site, as well as to the future of medical information on the Internet. With these results, the Department of Orthopaedics and Sports Medicine will be able to update and adapt its Web site based on actual users' needs. In addition, other organizations dedicated to providing online medical information can improve their Web sites' content and usefulness, based on this data about what people are seeking when they turn to the Internet for medical information. If health Web site managers can adapt their Web sites to meet their users' needs, patients may be able to find and use Internet-based health information more successfully. Finding relevant health information and support on the Internet may help people to become more actively involved in making decisions that affect their health, enabling them to participate more actively in their health care.

## Abbreviations

IP: Internet Protocol
